# Shut and re-open: the role of schools in the spread of COVID-19 in Europe

**DOI:** 10.1098/rstb.2020.0277

**Published:** 2021-07-19

**Authors:** Helena B. Stage, Joseph Shingleton, Sanmitra Ghosh, Francesca Scarabel, Lorenzo Pellis, Thomas Finnie

**Affiliations:** ^1^ Department of Mathematics, University of Manchester, Manchester, UK; ^2^ Emergency Response Department, Public Health England, London, UK; ^3^ MRC Biostatistics Unit, University of Cambridge, Cambridge, UK; ^4^ Laboratory of Industrial and Applied Mathematics, Department of Mathematics and Statistics, York University, Toronto Ontario, Canada; ^5^ CDLab - Computational Dynamics Laboratory, Department of Mathematics, Computer Science and Physics, University of Udine, Italy; ^6^ The Alan Turing Institute, London, UK

**Keywords:** COVID-19, school closure, school reopening, non-pharmaceutical interventions

## Abstract

We investigate the effect of school closure and subsequent reopening on the transmission of COVID-19, by considering Denmark, Norway, Sweden and German states as case studies. By comparing the growth rates in daily hospitalizations or confirmed cases under different interventions, we provide evidence that school closures contribute to a reduction in the growth rate approximately 7 days after implementation. Limited school attendance, such as older students sitting exams or the partial return of younger year groups, does not appear to significantly affect community transmission. In countries where community transmission is generally low, such as Denmark or Norway, a large-scale reopening of schools while controlling or suppressing the epidemic appears feasible. However, school reopening can contribute to statistically significant increases in the growth rate in countries like Germany, where community transmission is relatively high. In all regions, a combination of low classroom occupancy and robust test-and-trace measures were in place. Our findings underscore the need for a cautious evaluation of reopening strategies.

This article is part of the theme issue ‘Modelling that shaped the early COVID-19 pandemic response in the UK’.

## 1. Introduction

Throughout the course of the ongoing COVID-19 pandemic, the role of young people and children in transmission has been a question of particular concern [1,2]. This question is not only motivated by the goal of protecting the younger generations; it is also known from other respiratory diseases that, because younger people tend to have more prolonged and physical contacts among themselves [3], they pose a greater risk of infection to each other as well as being likely to introduce the infection to their respective households, and so can drive the epidemic [4,5]. Consequently, school closure is often one of the first measures considered when non-pharmaceutical interventions are needed [6]. However, during the COVID-19 pandemic it has often been implemented concurrently with other measures, making it difficult to assess its individual impact [7,8].

Many of the challenges inherent in quantifying the impact of closure remain when policy-makers subsequently turn to the reopening of schools. Reopening presents a myriad of further questions, such as the ages of those returning, the physical circumstances and timing of their return, and the necessary conditions that must be met on a community level before a return can be deemed safe enough. For new or emerging infections, answers to these questions require new efforts to establish an age-stratified understanding of the infection and transmission dynamics [9,10].

Earlier studies exist concerning the effectiveness of school closure as a means of controlling the spread of COVID-19, with mixed conclusions depending on the studied age group, country and modelling assumptions [6–9,11–13]. These sought to estimate the impact of school closure on nationwide transmission levels, be it, for instance, a reduction in the peak number of cases or the timing thereof. The challenges and questions related to school reopening have also been addressed from a theoretical or modelling perspective of scenario planning [14–16], estimating the number of new or severely ill cases resulting from school reopening. These studies use varying assumptions of the underlying community transmission, and consider various scenarios of the ages and timing of students returning to school.

While such models are a valuable means of quantifying the expected impact of measures without increasing the risk of exposing the wider community to infection, their inclusion of other interventions is necessarily limited. The times surrounding school closure and reopening have seen a myriad of other non-pharmaceutical interventions being implemented. Through social contact patterns, observed changes in community transmission are the result of non-trivial, underlying interactions between current interventions. We believe our work fills an important knowledge gap in the literature by addressing the context of school interventions alongside other measures, and the de facto impact of schools in a broader framework of non-pharmaceutical interventions.

School closure and reopening not only affect transmission occurring on school premises; they also affect (and are affected by) community transmission, transmission within households with young children, and wider measures taken to monitor and curb an outbreak. In addition to the well-being of children, school interventions also impact a nation’s workforce via the time dedicated to childcare. It must be remembered that the observed effects of these interventions are a product of underlying testing, reporting and isolation (or other physical or social distancing) measures.

The aim of this work is to carry out a comparative analysis of school interventions, making use of the diversity of available data streams, to serve as a complement to theoretical modelling efforts. Ours is a data-driven approach which does not seek to establish the individual role of school interventions on outbreak management, but instead assesses their impact in the context of wider societal interventions. Specifically, we wish to examine roles in transmission played by (a) the different age cohorts of students, (b) the timing of the school interventions (closure and reopening), and (c) the background or community incidence. We hope these results can serve as a series of lessons learned from nations that have already reopened schools.

For school closure, we address these questions by estimating the time between intervention and a response being observed in the recorded data, as well as the changes in the growth rate pre- and post-intervention. For school reopening, we track the growth rate in cases over the intervention timeline and search for correlations between these interventions and changes in the growth rate.

## 2. Methods

### (a) Data selection criteria

We consider four countries—Denmark, Germany, Norway and Sweden—owing to their geographical proximity, demographic similarities, and the relative timing and scope of their interventions which allow a better comparison. We distinguish between countries with medium-to-high (Germany and Sweden) and low (Denmark and Norway) levels of community transmission on the basis of daily COVID-19 cases, rather than cases *per capita* [17]. This is motivated by the feasibility of testing, tracing and isolating cases, which need not scale with population size. At the time of school closure, we saw the following cumulative cases: 800 (Denmark), 6500 (Germany), 1100 (Norway) and 1400 (Sweden). By the time schools started reopening, the total cases had risen to: 7000 (Denmark), 158 000 (Germany), 7200 (Norway) and 19 400 (Sweden on the day German schools first reopened). Only German states with at least 50 cases at the point of school closure, and at least 10 days of non-zero daily cases prior to closures, have been selected for analysis.

We considered hospital admissions as the primary data source in our analyses, where the numbers were available and sufficiently large to do so. All studied countries expanded their hospital surge capacities to accommodate patients to a sufficient degree that we are not aware of instances of COVID-19 patients being turned away. Since clinically ill patients are unlikely not to present themselves for treatment at hospital, admissions data are a practical measure of community infection and, unlike confirmed cases, are not as susceptible to variable testing rates in the wider population. However, by studying only a subset of the entire population, the data will be biased toward older and sicker individuals, which may for example lengthen the delay from an intervention to a visible signal in the data.

Confirmed cases were used in situations where hospitalization data were not available or were insufficient to reliably infer the effect of interventions—this was particularly relevant in the case of school reopening, which has predominantly been recommended in communities with significantly reduced daily incidence (and hence hospitalization) counts. Although we do not correct for variable testing rates in the confirmed cases, we have sought out datasets where there was evidence of consistent and thorough testing. However, we acknowledge this was a challenge for most countries in early March. We document the number of tests carried out and comment further on the reliability of confirmed cases as representative of the community epidemic in the electronic supplementary material (S3, Testing data).

Consistent test numbers for both Germany and Norway around the time of school reopening (see electronic supplementary material, S3) suggest that the confirmed number of cases is less prone to biases than earlier in the pandemic. We are therefore less concerned about using these data streams in a reopening context.

### (b) National data streams

The effect of school closure was estimated using hospitalization data for Denmark and Norway, and daily confirmed cases for Germany and Sweden. Given other interventions were implemented after school closure and their effect is hard to disentangle, it is implicitly assumed that the inversion of epidemic trend from growing to declining is not solely a result of school closure. Therefore, the analysis of the impact of school closure is restricted to data before the peak in reported cases or hospital admissions.

Denmark reopened schools quickly enough following sweeping nationwide interventions that hospitalization data could still be used, though we cross-checked these findings by analysing confirmed cases. Official estimates at the time suggest a delay from infection to hospitalization of 10–14 days [18]. Official Norwegian estimates suggest this same delay to be 14 days [19]. As Norwegian hospitalization data were too sparse to reliably infer the effect of school reopening, daily confirmed cases were analysed instead.

In Germany, daily confirmed cases are reported specifically for students under 18 in schools, kindergartens, holiday camps, after school clubs, etc., as well as for the staff working in these facilities. We used these numbers, and national hospital admissions, to analyse school reopening instead of population aggregates on the state or federal level.

References for each data stream, together with further discussion on their limitations, are provided in the electronic supplementary material (S1, Data availability).

### (c) Estimating the effect of school closure by simulating the early epidemic

Our aim is to assess the impact of school closure on transmission dynamics. This includes any associated changes in behaviour (e.g. parents not accompanying children to school or working from home owing to caring responsibilities), as well as all other interventions (if any) occurring on the same day, which cannot be disentangled from school closure. The impact is assessed by comparing differences in the growth rate of cases or hospitalizations before and after the intervention.

Rather than naively comparing the growth rate at different points in time, we follow a more sophisticated procedure with the aim of separating the decrease in growth rate due to interventions implemented before school closure, and the impact of school closure itself. This is achieved by generating a *counterfactual* projection of daily cases or hospital admissions, which accounts as much as possible for events prior to, but excluding, school closure, and identifying when there is a clear deviation between the real data and such a projection. We then compare the growth rates observed in the real data and in the modelled counterfactual at the time when this deviation is detected, and interpret this difference as the likely impact of school closure.

To construct the counterfactual, we use a compartmental ordinary differential equation (ODE) model fitted to the pre-intervention data. However, fitting a model to data from the earlier part of the epidemic is extremely challenging since the observations are generally scarce, noisy and coloured through various reporting issues, in particular systematic ones, such as a strong weekend effect.

Fitting a simple compartmental model without accounting for these factors will result in parameter estimates that are systematically biased [20]. These inaccuracies in parameter estimates propagate to any projection drawn from the model. Mitigating this model discrepancy in the fitting process is an area of active research; see [21] for a recent review. Generating a counterfactual projection using a compartmental model, without compensating for such discrepancies, will erroneously understate or overstate the effect of the intervention.

To alleviate the challenges brought on by scarce and noisy data we argue that, given the 4.8-day mean incubation period for SARS-CoV-2 [22], we expect the impact of any intervention to be delayed by at least 5 days, and in particular we expect cases on the first 5 days following school closure to predominantly reflect only earlier interventions (whether imposed or not, e.g. spontaneous physical distancing). We then use a two-step approach for fitting an ODE model and correcting for the discrepancy between model and data as follows. In the first step, a selection of sample trajectories are generated via approximate Bayesian computation (ABC) fitting of the ODE from the first day of data until 5 days after school closure. In a second post-processing step, a Bayesian regression model is then trained on the same data used to fit the ODE model, while using the sampled trajectories as the covariates (inputs). Essentially, the regression model attempts to capture the structural part of the discrepancy between ODE simulations and the observed data (predominantly, potential deviation from exponential growth and weekend effects). We formulate this regression model as a Gaussian process (GP) with a negative binomial likelihood. Once trained, the regression model is used to project the trajectory of cases for the time period following the 5 days after school closure. This projection is then treated as the desired counterfactual.

We identify the first day on which there is a clear and sustained deviation from the modelled data, hereafter referred to as the *response date*. Such a deviation must (a) occur more than 5 days after the date of school closure, (b) fall outside of the 75th percentile of the projected data, and (c) persist in doing so for at least 5 days. The time window from school closure to response date defines the *lag time* (table 1, column 2), which runs from the date of closure (acting as the zeroth day) up to but not including the response date.

**Table 1.** Estimated lag time and pre- and post-intervention (and for the latter, modelled and observed) daily growth rates in different German states, and relative change between the modelled and observed growth rate. The 95% credible intervals (CrI) are given in parentheses. Their equivalent formulation as doubling times can be found in the electronic supplementary material (electronic supplementary material, table S3). Sensitivity analysis of the training period on the lag time suggests these can vary by up to 2 days (see electronic supplementary material, S2.7). The overlapping CrIs between the pre-intervention and the post-intervention modelled growth rates suggest a limited deviation from exponential growth between the day of school closure and the end of the training window.

state	lag time (days)	$r_{\;\mathrm{pre}}^{\mathrm{obs}}\;(d^{-1})$	$r_{\;\mathrm{post}}^{\mathrm{mod}}\;(d^{-1})$	$r_{\;\mathrm{post}}^{\mathrm{obs}}\;(d^{-1})$	$1-r_{\;\mathrm{post}}^{\mathrm{obs}}/r_{\;\mathrm{post}}^{\mathrm{mod}}$
Baden-Württemberg	8	0.143	0.167	0.051	69%
		(0.104–0.182)	(0.148–0.185)	(0.013–0.089)	(40–93)
Bavaria	8	0.216	0.214	0.109	49%
		(0.176–0.255)	(0.208–0.221)	(0.072–0.146)	(29–67)
Berlin	—^a^	0.145	—	—	—
		(0.103–0.187)			
Hesse	7	0.251	0.274	0.067	75%
		(0.195–0.308)	(0.265–0.283)	(0.017–0.117)	(56–94)
Lower Saxony	7	0.223	0.229	0.069	70%
		(0.179–0.267)	(0.213–0.244)	(0.032–0.107)	(50–87)
North Rhine-Westphalia	6	0.192	0.206	0.061	70%
		(0.156–0.228)	(0.200–0.213)	(0.026–0.096)	(52–88)
Rhineland-Palatinate	7	0.257	0.235	0.043	82%
		(0.205–0.310)	(0.211–0.259)	(0.001–0.086)	(59–100)

^a^The peak in daily incidence is reached before a response is seen in the data. A lag time that may be attributable to school closures therefore cannot be determined.

The growth rates are obtained as point estimates (see following description of the instantaneous growth rate) at the time of school closure, for the observed data ($r_{\;\mathrm{pre}}^{\mathrm{obs}}$, table 1, column 3), and at the response date for both the modelled ($r_{\;\mathrm{post}}^{\mathrm{mod}}$, table 1, column 4) and the observed data ($r_{\;\mathrm{post}}^{\mathrm{obs}}$, table 1, column 5). The relative changes in the estimated growth rates between modelled and observed data at the response date can be used to assess the impact of school closure. The observed growth rate at the time of school closure can be used to cross-check the growth rate in the modelled data; these could be significantly different if the impact of interventions prior to school closure had a strong effect on transmission which the GP was able to capture in the counterfactual projection. However, a causal link cannot be established between the interventions and the growth rates, calling for a cautious interpretation of the specific values of the growth rates and the reductions therein.

The ABC fitting of the SEIR model was carried out using the PyGOM package v. 0.1.6 for Python [23]. The GP regression method, devised as a Bayesian latent variable approach, was carried out using the PyMC3 probabilistic programming package for Python [24]. Further details about the introduced methods can be found in the electronic supplementary material (S2, Numerical methods).

### (d) Estimating the effect of closure and reopening using the instantaneous growth rate

With the number of sequential changes in interventions and loosened restrictions on personal movement and the operation of businesses, it is misleading to estimate a constant growth rate in new cases before and after schools reopened. We therefore consider a method whereby the growth rate can be quantified following successive changed measures. A smoother *ρ*(*t*) is applied to the data over time *t*, such that the instantaneous growth rate is *ρ*′(*t*) (cf. a constant value in a phase of pure exponential growth or decline). It is assumed that the daily new confirmed cases (or daily new hospital admissions) *c*(*t*) grow or decay exponentially, with noise added to account for small case numbers, i.e. *c*(*t*) ∝ e^*ρ*(*t*)^. To estimate *ρ*′(*t*), we adapt a general additive model (GAM) from the R package *mgcv*, using a negative binomial family with canonical link [25]. Smoothing is achieved using default thin plate regression splines.

Where case numbers are sufficiently high, and there is a clear weekend effect in the reporting of data, a weekend effect has been accounted for in the GAM by the addition of a fixed effect on those days of the week. Specifically, we add a quantity *ω*_*d*_ for each day of the week *d* ∈ [1, 7], such that cases follow $c(t)\propto e^{\omega_{d}+\rho (t)}$. Setting *ω*_*d*_ = 0 for all but 2 days yields a weekend-specific description, and $\omega_{d}=0\forall d$ recovers the GAM with no day-of-week effects. This method has previously been used in [22].

In the case of school closure, the GAM approach with a weekend effect has been used to estimate the instantaneous growth rate at different points in time. In the case of school reopening, the instantaneous growth rate with a day-of-week effect has been used to identify trends in the data. Our aim here is to assess if there is any correlation between changes in the growth rate, and the timing of school reopening.

## 3. Results

As many of our findings are based on the premise of analysing interventions at different points in time, or in different geographical regions, all results are inherently conditional on the assumptions of *stability* and *homogeneity*. Firstly, in order to make comparisons throughout the same time series, we assume that the only changes to behaviour are those directed by changing public guidelines, and that adherence to these is constant throughout (stability). Secondly, in order to compare different regions of the same country, we must assume that there are no fundamental differences in adherence, testing, implementation of national policies or similar such aspects (homogeneity). Deviations from these assumptions are taken to be too small to affect the data in a way to qualitatively alter our conclusions.

### (a) Closing of schools in Germany

We consider the date of school closure as the first day on which all schools in a state were closed as a response to state or national government intervention. In most cases, however, there were local school closures prior to enforced closures. Furthermore, most primary schools continued to be open to both vulnerable children and the children of key workers after national and state closures.

Table 1 provides an overview of the observed changes in the daily growth rates in the period during and after school closures. These growth rates are consistent with previous estimates [26].

All states in Germany saw a reduction in growth rate after the closure of schools, typically after a delay of 7 days, or about 1.2 generations [27]. With the exception of Baden-Württemberg and Berlin, all German states closed schools on 16 March. As this was a Monday, we set the effective date of school closures as Saturday 14 March, under the assumption that school activity is significantly reduced on weekends. Schools in Baden-Württemberg and Berlin closed on Tuesday 17 March. It should be noted that all states experienced further interventions around the same time as school closures. The presence of concurrent interventions makes it difficult to attribute the fall in cases solely to the closure of schools, and it is likely that a combination of factors contributed to the observed decay in growth rate. However, comparison between Baden-Württemberg and North Rhine-Westphalia, which saw similar case numbers, yields comparable lags and overall trajectories of the epidemic curves when accounting for the 3-day delay in school closures in Baden-Württenberg (see electronic supplementary material, figure S9). This is indicative of school closures being at least partially responsible for the reduction in growth rate.

The reduction between the modelled and observed post-response growth rates serves as a measure of the overall effectiveness of interventions (table 1, column 6). Overall, lower relative reductions in the growth rate are weakly correlated with states that had higher (daily and cumulative) incidence counts at the time of intervention (Baden-Württemberg, Bavaria and North Rhine-Westphalia). This supports the generally held expectation that non-pharmaceutical interventions are more effective when implemented early, capable of breaking transmission chains while community transmission is relatively low.

**Figure 1.**
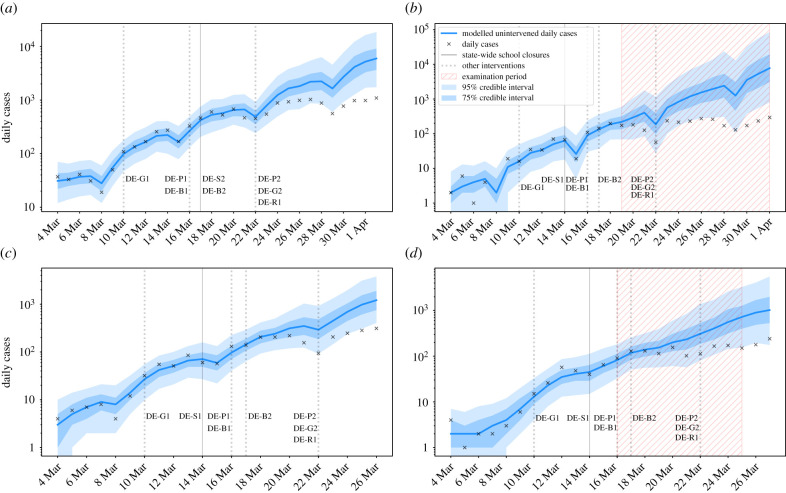
Modelled and observed cases in (*a*) Baden-Württemberg, (*b*) Hesse, (*c*) Lower Saxony, and (*d*) Rhineland-Palatinate. Hesse and Rhineland-Palatinate (*b*,*d*), where final year high school exams took place in late March, saw a similar response to interventions to other German states with moderate incidence (*c*) where exams did not take place at that time. While there is insufficient scope in the data to assess the effect of the full examination period, we should in principle be able to detect a signal related to the beginning of the examination period. Assuming stability and homogeneity across states, and given the lack of such a signal, it is unlikely that these exams significantly contributed to the overall outbreak. (Online version in colour.)

The states of Hesse and Rhineland-Palatinate allowed students aged 18–19 to sit in-school examinations in late March, under strict social distancing measures and other precautions. Neither of the states permitting examinations saw any less evident reduction in growth rates compared with states that had similar case numbers prior to school closure but where exams did not take place during this time period (e.g. Lower Saxony). Further, the largest reduction in the growth rate was observed in Rhineland-Palatinate. Assuming stability and homogeneity, this suggests that under controlled conditions with limited social interaction, the beginning of the examination period for older students was likely not a significant driver of epidemic growth. We cannot comment on the full effect of the entire examination period. We include the detailed results from the highlighted German states in figure 1 and a timeline of key interventions from [28] below, with the remaining states detailed in the electronic supplementary material (S4, School closures analyses).

— 10 March – Ban on gatherings of more than 1000 people (DE-G1).
— 14 March – **Hesse, Lower Saxony, and Rhineland-Palatinate closed schools** (effective date, DE-S1).
— 16 March – Borders shut with France (FR), Switzerland (CH), Austria (AT), Denmark (DK) and Luxembourg (LU) (DE-B1); closure of non-essential businesses and public services (DE-P1).
— 17 March – **Baden-Württemberg closed schools** (DE-S2); shut borders with EU (DE-B2).
— 22 March – National stay-at-home order, with exceptions for essential trips, and ban on gatherings of more than two people (DE-G2); ban on all social events and gatherings (DE-P2); closure of non-essential retail and leisure, with exceptions for restaurant takeout (DE-R1).

### (b) Closing of schools in Denmark, Norway and Sweden

In all three countries, there were provisions in place to allow key workers’ children to continue attending school. Hospital admissions were analysed for Denmark and Norway, as testing was deemed too variable during this time period (see electronic supplementary material, S3) to reliably use confirmed cases. However, the expected lag time from infection to hospital admission in Denmark and Norway is 10–14 days [18,19], whereby any signal observed in the data is too early to be attributable to school closures. For completeness, we include the fits to daily hospital admissions in the electronic supplementary material (S4, School closure analyses).

**Figure 2.**
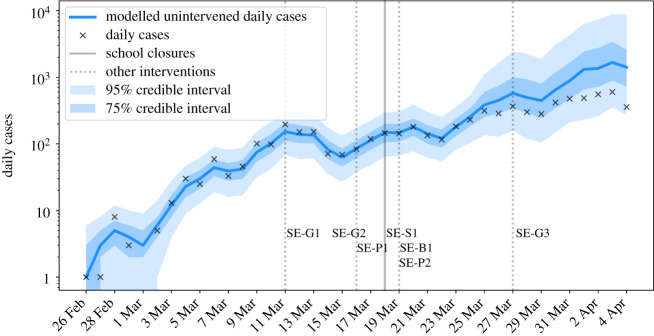
Modelled and observed daily cases in Sweden. (Online version in colour.)

Sweden’s school closures were less restrictive than other countries’, with only educational establishments for students aged 16 or over being required to close. Despite no official nation wide closing of primary or secondary schools in Sweden, there were local closures in response to outbreaks within the community. There is no evidence of a sustained reduction in the growth rate within a time period attributable to school closures (figure 2). It is notable, however, that the limited closures in Sweden were imposed in the absence of large-scale social restrictions. This indicates that school closures affecting older students without more widespread social interventions are unlikely to have significant national effects, and that school closures are most effective when implemented concurrently with other interventions.

It is notable that there was an increase in weekly testing between 30 March and 6 April, which may have contributed to the apparent limited reduction in growth rate during this time. However, this falls outside of the time window in which we would expect to see a response attributable to school closures.

Sweden saw the following interventions introduced around the same time as school closures:

— 11 March – Ban on gatherings of more than 500 people (SE-G1).
— 14 March – Advice against non-essential travel (SE-G2).
— 16 March – Social distancing advised but not enforced (SE-P1).
— 18 March – **Closure of all education for students aged 16 or over** (SE-S1).
— 19 March – Restrictions on international travel (SE-B1); advice against national travel (SE-P2).
— 27 March – Ban on gatherings of more than 50 people (SE-G3).

### (c) Reopening of schools

#### (i) Germany

The following key interventions, sourced from [28], are possible confounders for the effects of school reopening:

— 20 April – Opening of some retail venues (DE-R2).
— 22 April – Mandatory mask wearing in certain public spaces (DE-P3).
— 27 April – **Return of Year 10, final year exam students (ages 15, 18–19)** (DE-S3).
— 29 April – Extension of mask-wearing requirements (DE-P4).
— 3 May – Expiry of stay-at-home order (DE-G3).
— 4 May – **Return of Year 4 primary school students (age 9)** (DE-S4); opening of retail (DE-R3) and public spaces (DE-P5).
— 11 May – **Return of primary and secondary school students (ages 9, 15, 17–19)** (DE-S5).
— 15 May – Relaxation of border controls (DE-B3).
— 18 May – **Staggered return of primary and secondary school students (ages 9–11, 15–19)** (DE-S6); meeting of two households allowed (DE-G4).
— 29 May – Gatherings of up to 10 people allowed (DE-G5).
— 2 June – Pubs reopened (DE-R4).

Owing to differing policies across German states, the dates of school reopening and the ages of students returning were variable. Where an age group appears over multiple dates, the return of students in this age group took place in different states. We present a summary of the overall national trend, as our data only distinguish between staff and students on the national scale. On three occasions the recorded cases were inconsistently reported, and values were imputed using cases reported on preceding and proceeding days. Our findings do not change significantly upon exclusion of these data points. We contrast these demographically specific findings by comparison with national hospital admissions.

**Figure 3.**
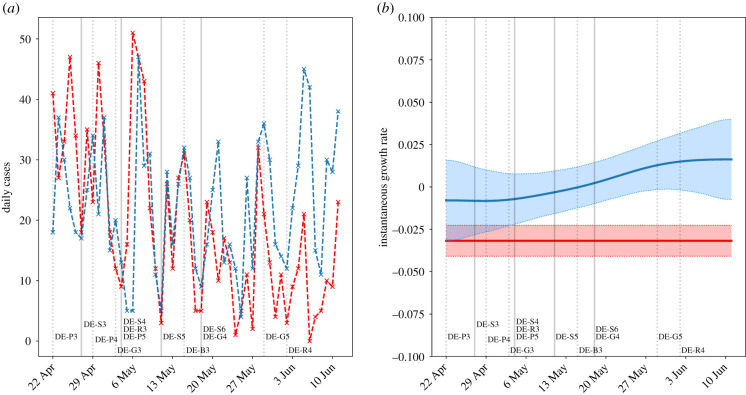
Confirmed cases in staff (red) and students (blue) in schools, kindergartens, holiday camps and other educational facilities for under-18s (age distribution not known) in Germany. (*a*) Daily new confirmed cases; (*b*) instantaneous growth rate (shaded regions are 95% confidence intervals). Solid vertical lines indicate when students returned to school, and dashed lines indicate other changes to public measures. In April and early May with small numbers of primary school or exam students returning, there was no notable difference between the incidence among students and staff. Accounting for the lag time, the incidence among students was higher than that of staff following 18 May.

The spike in daily cases observed around 7–8 May (figure 3*a*) may be a result of increased presentation for testing following a national announcement of school reopening on 4 May (allowing for testing delay), or increased community transmission following reopening of other parts of society which was subsequently contained. Overall the incidence among staff decreased, which is supported by the growth rate among staff being negative. The incidence among students first decreased, and subsequently increased, with a predominantly positive growth rate from the end of May (figure 3*b*).

The stable, low, values of the incidence and growth rate until the middle of May indicate that the return of final year and Year 4 students either (a) did not significantly increase transmission in schools or the community, or (b) did increase transmission, but this was mitigated owing to safety protocols of prevention and monitoring. This observed effect is quite a strong signal as the daily case counts remain low even across a background of increased community transmission from late April onward with, for example, shops reopening. It is therefore reasonable to conclude that these age groups do not strongly increase transmission in a setting of effective social distancing.

**Figure 4.**
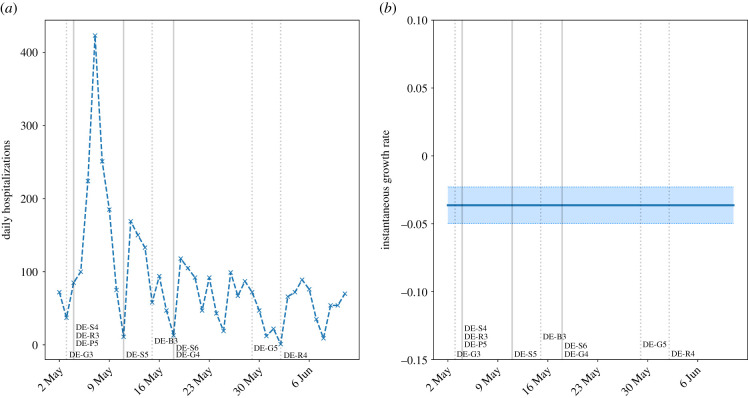
Daily hospital admissions with COVID-19 in Germany, excluding those working in education, front-line healthcare workers, carers, catering and hospitality, thus representing transmission in the general, average-exposure population. (*a*) Daily admissions; (*b*) instantaneous growth rate (shaded regions are 95% confidence intervals). The continuing decline in admissions suggests that the return of younger (and exam) students did not present a statistically significant impact on the general hospitalized population. It is worth bearing in mind that hospital admissions lag further behind than confirmed cases. Additionally, since very few young people have been hospitalized, an additional generation time of 6 days [27] may need to be added to this lag to account for students infecting older age groups. (Online version in colour.)

However, the impact of most students returning to school from late May was different. In this time period, the incidence among staff qualitatively agreed with the national trend in hospitalizations (figure 4), i.e. staff did not immediately appear to be at greater risk following the return of more students. By contrast, the growth rate in student cases increased following 18 May. The constant staff growth rate, and the small effect of the return of (mostly) younger years, suggest that the increased incidence may be due to (a) increased transmission among older students, (b) low feasibility of effective physical distancing in venues at full capacity, or (c) a combination of these.

#### (ii) Denmark

Schools reopened alongside the following key interventions (sourced from [28]):

— 8 April – 7-day ban on gatherings of over 10 people (DK-G5).
— 14 April – Partial return of employees to work (DK-P2).
— 15 April – **Return of nursery, kindergarten, Years 0–5 primary school, and final year exam students (ages 0–12, 18–19)** (DK-S3).
— 20 April – Partial reopening of retail and small businesses (DK-R2).
— 21 April – Assemblies limited to 500 people (DK-G6).
— 11 May – Full reopening of shopping and retail (DK-R3).
— 18 May – **Return of Years 6–10 secondary school students (ages 12–16), and examinations requiring physical attendance** (DK-S4); restaurants and cafés reopen (DK-R4); reopening of houses of worship (DK-P3).
— 21 May – Reopening of leisure and cultural facilities (DK-P4).
— 25 May – Relaxation of border restrictions with Nordic countries and Germany (DK-B2).
— 27 May – **Return of secondary school students (ages 16–18) and adult education** (DK-S5).

**Figure 5.**
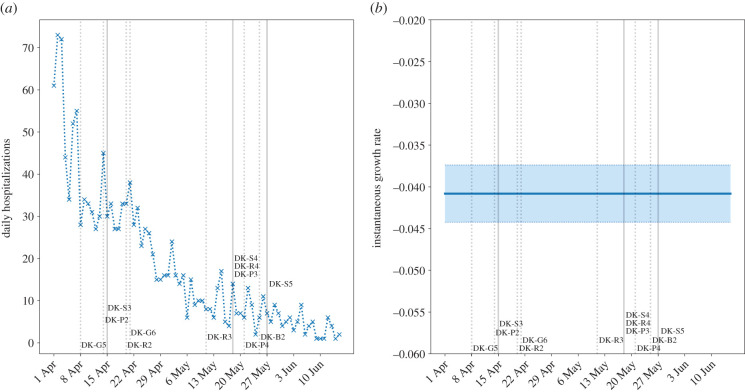
Daily hospitalizations with COVID-19 in Denmark. Admissions are shown in (*a*), and (*b*) shows the instantaneous growth rate (shaded regions are 95% confidence intervals). A longer lag time of 10–14 days is in effect from infection to hospitalization [18], with a further 6 days’ generation time [27] to account for subsequent infection generations owing to the low hospitalization rate among children. Solid vertical lines indicate when students returned to school, and dashed lines indicate other interventions. (Online version in colour.)

There is no significant observable increase in the growth rate of hospital admissions following school reopening to younger years, even bearing in mind the subsequent reopening of some businesses (figure 5). The low growth rate and small relative number of admissions suggest that the return of younger years to school with social distancing did not contribute significantly to community transmission. The subsequent reopening stage on 18 May also did not have a significant impact on hospital admissions, which we verify using confirmed cases (see electronic supplementary material, S5, School reopening analyses).

These findings are further supported by a lower proportion of adults testing positive for COVID-19 among those working with children aged 0–16 than those working with students aged 16 or over (see electronic supplementary material, S5) [29]. However, these numbers alone do not distinguish between infection acquired from students and infection acquired elsewhere.

#### (iii) Norway

The following events are possible confounders in the data, and key dates for school reopening (sourced from [28]):

— 1 April – Exceptions made to entry restrictions (NO-B4).
— 8 August – Easing of entry restrictions from European Economic Area (EAA) workers (NO-B5).
— 20 April – **Return of kindergarten students (ages 1–5)** (NO-S2); travel to cabins allowed (NO-G5).
— 27 April – **Return of Years 1–4 (ages 6–10) and final year students (ages 18–19), vocational training, and higher education requiring physical attendance** (NO-S3); partial reopening of retail and small businesses (NO-R1).
— 7 May – Events, and some public sports and leisure facilities open, but limited to 50 people (NO-P1); group size for social gatherings increased from 5 to 20 people (NO-G6).
— 11 May – **Return of students aged 10–18 this week** (NO-S4); reopening of bingo halls and driving schools (NO-P2).
— 12 May – Easing on entry restrictions (NO-B6).

**Figure 6.**
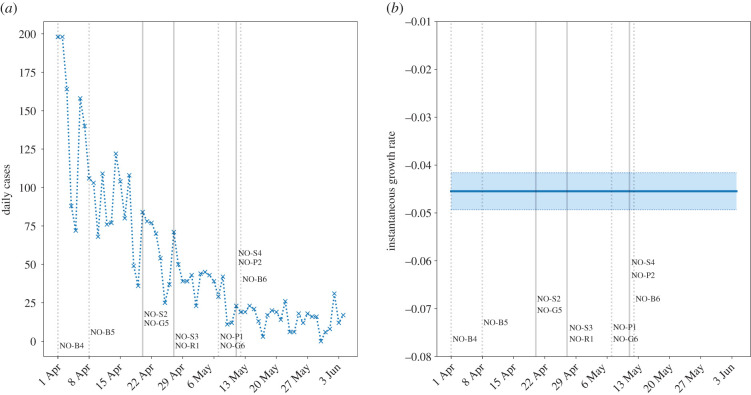
Daily confirmed cases in Norway. (*a*) New cases; (*b*) instantaneous growth rate (shaded regions are 95% confidence intervals). Solid vertical lines indicate when students returned to school, and dashed lines indicate other interventions. (Online version in colour.)

There is no notable change in the growth rate, even following the return of students in May (figure 6). The consistently negative growth rate and small number of cases indicate that the return of most students to school (with social distancing) did not contribute significantly to community transmission. However, this effect is likely conditional on high levels of testing, with very low community transmission.

## 4. Discussion

Decoupling the effect of school closure, and subsequent reopening, from other interventions is not straightforward. This work does not claim to have achieved this; however, the consistency of the signal across regions with different intervention timelines suggests a distinct effect of school closure on the subsequent growth in cases. The consistently lower post-intervention growth rates in German states when compared with the modelled scenarios with no interventions (table 1) suggest that school closures contributed to reducing the epidemic growth rate. School interventions were typically followed by a response in the data approximately 7 days later (corresponding to approx. 1.2 generations). Table 1 shows that this lag time was comparable across states that closed schools at different times. High school students sitting their final examinations under social distancing does not appear to have significantly impacted case numbers. Sweden implemented partial school closures that affected students aged 16 or above. However, there is no evidence to suggest that this intervention affects the later daily incidence within the expected time frame. These findings are consistent with earlier works suggesting that school closure in isolation is insufficient to prevent the spread of COVID-19 [6,8,11]. The evidence for the impact of school closures on growth rates in Norway and Denmark is more limited. While there was a reduction in growth rate of hospitalized cases after school closures, it has not been possible to link this effect with school closures.

While school closures are often among the first implemented control measures, school reopenings are typically staggered with other eased restrictions, often with a small initial cohort of returning students. Since, to our knowledge, no stringent restrictions were introduced to compensate for the additional transmission risk due to school reopening, a lack of signal in the growth rate after reopening would be indicative that schools do not contribute substantially to community transmission. From our analysis, the reopening of schools to younger year groups and exam students in Germany, Denmark and Norway has not resulted in a significant increase in the growth rate. The return of all students (up to age 16 in Denmark) does not appear to have increased transmission in Denmark and Norway. However, the added return of most (primarily older) students in Germany has increased transmission among students, but not staff. It is unclear whether older students transmit more, or if physical distancing is practically unfeasible in classrooms at high capacity. The distinction between the impact of younger and older students is echoed in other modelling studies [14,15]. Although our findings cannot be interpreted as causal links between individual interventions and changes in national case numbers, they represent a realistic assessment of the effects of school reopening in their natural context of wider societal interventions.

Our findings are not without limitations. The presence (or lack) of signals in the data following school interventions is limited by the reliability of the available data. We have worked with hospital admissions data, as they are less affected by variable testing, while bearing in mind that hospitalizations only affect a subset of the infected population. Where these data were unavailable, we have considered confirmed cases while monitoring the degree of testing in place to ensure such numbers were indicative.

The GP regression method allows one to account for differences between the simulated epidemic trajectories from the ODE model and the observed cases. However, the fact that closures occurred very early on in the epidemic means that the GP method often had to be trained on a limited number of data points.

Since the instantaneous growth rate relies on the derivative of splines, it is subject to increased error at the boundaries of the data. However, the observed signals are qualitatively robust to this limitation. Owing to the noisiness of some data streams from relatively low incidence following mass quarantine, the values of the instantaneous growth rate should be taken as a quantification of the trend in incidence rather than the true value on any given day.

The data have generally not made it possible to account for inevitable geographic variability, the age distribution of those studied, and their occupation (i.e. likelihood of exposure to infected individuals) in our analyses. The analysis of German school reopening, particularly the comparison of staff and student infections, warns against the reliability of using national-level data to understand the immediate effects or impact on a single population. Rather, such impact may only become visible in national data in subsequent generations. We must therefore be concerned not only with the lag time from intervention to a signal in the data, e.g. 7 days in Germany, but also with the following generation of infections.

Our analysis is restricted to countries with high monitoring and intervention efficacy (including but not limited to high testing, tracing, and adherence to isolation), hence great care should be taken when translating our findings on the impact of school reopening to other nations. For instance, continued low incidence following the return of younger students does not imply that such a measure is inherently safe, but may rather be a result of the successful implementation of a complete system of monitoring and interventions jointly with low daily incidence, as observed in Denmark and Norway. In many instances, the students were spread over more classrooms, with greater levels of physical distancing from each other and teachers, conditions that are not always practically feasible.

Caution is warranted surrounding the return of older students, in particular regarding the likely increased number of crowded classrooms, as well as their added impact to community transmission. The correlation with community transmission is particularly clear in Germany, with confirmed cases increasing among students, and the halted decay in hospital admissions on the national level. While all three countries seem to be effectively managing transmission, the volatility of new German hospital admissions warns that a failure in control, or a sudden spike in cases, will likely have a stronger effect in Germany than it would have in Denmark or Norway. Key to this observation is the aforementioned delay before which the ripple effects of school reopening will travel from students to the general population. Furthermore, we highlight that the tenuous balance (net zero growth in June) in Germany exists despite a swift and robust test and trace infrastructure and school-level stratified monitoring. We question the possibility of an equally effective reopening in countries with a monitoring process reliant on national-level incidence data. The swiftness and effectiveness of targeted interventions become increasingly crucial as the daily incidence increases, owing to the correspondingly greater challenges presented in managing the localized outbreaks across e.g. reopened schools.

Policy-makers should carefully consider their nations’ respective capacities and associated effectiveness of infection management before considering a partial or full reopening of schools. Our findings suggest a small, strategically chosen, proportion of students should return in the first instance, with dedicated testing and monitoring of cases among staff and students (over time scales where the effect can be assessed). Any significant return of students to schools, particularly in countries with a high incidence, should not be considered unless an infrastructure is in place that would be able to swiftly identify and isolate most new cases as they appear [16]. Such a strategy may not be feasible if the community incidence is too high.

When used in conjunction with household transmission models, and knowledge of the length of immunity associated with SARS-CoV-2, our findings may be used to inform age-targeted vaccine allocation protocols.

## 5. In context

Key findings of this paper, including the disparate effects of school interventions on cases among staff and students, were first sent to SPI-M on 10 June, and presented on 17 June 2020. An early version of the analysis was published on medRxiv on 25 June 2020. In conjunction with other modelling work on school interventions, this work contributed to the knowledge base upon which recommendations pertaining to school closure and reopening were based throughout the pandemic. Since the writing of this manuscript, a growing body of work on school interventions has accumulated, highlighting the need for monitoring via age stratified case data, dedicated testing in schools and distinguishing between older and younger students. The findings reflect transmission patterns in early 2020, and so do not account for vaccinations in the population, nor the presence of more transmissible variants. Care should, therefore, be taken when applying the results in a different context.

## Data accessibility

All data used in the production of this work, including the relevant scripts, can be found in the electronic supplementary material and online at https://github.com/HelenaStage/COVIDschools.
